# Supplementary material for: Computational model of alpha 7 nicotinic acetylcholine receptor in thalamic reticular nucleus neurons and their involvement in network states
Source: PLoS One. 2025 Aug 29;20(8):e0330635. doi: 10.1371/journal.pone.0330635 (PMC12396715; doi:10.1371/journal.pone.0330635)
Supplement: File S1 — Table 1 – Parameters. Table 2 – Initial Values. (DOCX) [file pone.0330635.s001.docx]

**Supplementary**

**Supplementary Methods: Computational Model Experiment**

Network reverberations in the model were initiated through a 100 ms current injection to the thalamic reticular nucleus (TRN) cell model. Interspike times (ISTs) were recorded and subsequently inverted to produce a list of interspike rates for the 20 seconds following the stimulus. These interspike rates were then used to generate histograms, which are presented in the 1st, 3rd, and 5th panels of **Figure 7D**. The resulting interspike rate data, spanning 20 seconds, provides insights into the frequency composition of the model, mirroring brain-like dynamics.

To compare frequency compositions resulting from variations in model parameters, histograms were presented as heat maps, with color representing the number of occurrences. Interspike frequencies are arranged vertically, while model parameters are presented horizontally. The parameters were organized within a nested loop structure to capture their hierarchical impact on network dynamics.

The outermost loop is defined by the α7 conductance parameter, which steps through seven values: 0%, 50%, 75%, 90%, 100%, 110%, and 125%. This outer loop produces the seven columns displayed in **Figure 7C**. The remaining parameters are arranged based on their relative impact on the network’s dynamics, with parameters having the least effect being looped most frequently. This organization allows for the identification of qualitatively conserved dynamics across different parameter configurations.

For example, the lower panel of **Figure 7B** shows frequency composition for the default α7 conductance value, focusing on frequencies up to 45 Hz. Here, two distinct dynamical states are apparent: one with a broad and constant frequency composition over the first third of the parameters, and another with narrower, lower frequency bands in the latter two-thirds. These variations in frequency composition can provide insight into the underlying mechanisms of the network’s behavior.

Moreover, the parameters are organized to reflect a transition from wake-like to sleep-like promoting effects, which we define as the *sleep-axis*. The least influential parameter, *rebound excitation duration*, was looped the most often. This parameter was tested at three values (320 ms, 80 ms, and 20 ms), and it was hypothesized that reducing the rebound excitation duration would result in decreased network activity, thereby promoting sleep-like states. After each iteration of this loop, the *rebound excitation strength* parameter was updated, stepping through values of 7.0, 3.5, and 1.75 µA/cm². Following this, the *stimulation delay* parameter was adjusted through values of 20 ms, 40 ms, 80 ms, and 160 ms. It was anticipated that weaker and delayed excitation would further facilitate sleep-like states.

The final parameter before α7 conductance was the *adenosine parameter*, modeled by stepping the potassium leak conductance through three values: 0.07154, 0.14, and 0.22062 mS/msec/cm². The parameters were arranged along the sleep-axis so that persistent low and high-frequency bands could be detected across some, but not all, parameter changes, enabling the identification of mechanisms that influence transitions between wake- and sleep-like dynamical states.

Cross-correlation analyses were performed on the spike-rate histograms to identify network states with similar distributions of interspike rates. The zero-lag Pearson correlation coefficient was used to quantify the degree of correlation between histograms. This cross-correlation analysis followed the same sleep-axis organization as the histograms, facilitating the identification of specific patterns of network activity across different model configurations.

**Membrane Potential and Ionic Currents –**

$\frac{{dV}_{m}}{dt}=-\frac{1}{C_{M}}\left( I_{Na}+ I_{K}+ I_{Cl}+ I_{T}+{C_{M}(I}_{NCX} \right)+I_{KCa}+I_{CAN}-I_{app}$ 1. (Membrane Potential)

$I_{Na}=g_{Na}\left( m_{H}^{3} \right)\left( h_{H} \right)\left( V_{m}-E_{Na} \right)+ g_{NaL}\left( V_{m}-E_{Na} \right)$ 2. (Na^+^ Current, Fast/Leak)

$I_{K}=g_{K}\left( j_{H}^{4} \right)\left( V_{m}-E_{K} \right)+ g_{KL}\left( V_{m}-E_{K} \right)$ 3. (K^+^ Current, Fast/Leak)

$I_{ClL}=g_{ClL}\left( V_{m}-E_{Cl} \right)$ 4. (Cl^-^ Current, Leak)

$I_{T}=g_{TCa}\left( m_{Ca}^{2} \right)\left( h_{Ca} \right)\left( V_{m}-E_{Ca} \right)$ 5. (T-type Ca^2+^ Current)

$I_{NCX}=\left( \frac{I_{NCXmax}\left[ {\exp\left[ \frac{\left( \gamma\right)VF}{RT} \right][{Na}^{+}]}_{i}^{3}{[{Ca}^{2+}]}_{o}- {\exp\left[ \frac{\left( \gamma-1 \right)VF}{RT} \right][{Na}^{+}]}_{o}^{3}{[{Ca}^{2+}]}_{i} \right]}{(K_{m,Na}^{3}+\left[ {Na}^{+} \right]_{o}^{3})(K_{m,Ca}+\left[ {Ca}^{2+} \right]_{o})(1+k_{sat}\exp\left[ \frac{\left( \gamma-1 \right)VF}{RT} \right])} \right)$ 6. (Na^+^/Ca^2+^ Exchanger Current)

$I_{KCa}=g_{KCa}\left( m_{KCa}^{2} \right)\left( V_{m}-E_{K} \right)$ 7. (Ca^2+^-dependent K^+^ Current)

$I_{CAN}=g_{CAN}\left( m_{CAN}^{2} \right)\left( V_{m}-E_{CAN} \right)$ 8. (Ca^2+^-dependent Nonspecific Cation Current)

$I_{NaCAN}=g_{NaCAN}\left( m_{CAN}^{2} \right)\left( V_{m}-E_{Na} \right)$ 9. (Ca^2+^-dependent Nonspecific Cation Current, Na^+^ part)^[[1]](#footnote-1)^

$I_{KCAN}=g_{KCAN}\left( m_{CAN}^{2} \right)\left( V_{m}-E_{K} \right)$ 10. (Ca^2+^-dependent Nonspecific Cation Current, K^+^ part)^1^

**Activation and Inactivation Variables –**

$\frac{{dm}_{H}}{dt}= \alpha_{m}\left( 1-m_{H} \right)-\beta_{m}m_{H}$ 11. (Na^+^/K^+^ Current, Fast/Leak)

$\frac{{dh}_{H}}{dt}= \alpha_{h}\left( 1-h_{H} \right)-\beta_{h}h_{H}$ 12. (Na^+^/K^+^ Current, Fast/Leak)

$\frac{{dj}_{H}}{dt}= \alpha_{j}\left( 1-j_{H} \right)-\beta_{j}j_{H}$ 13. (Na^+^/K^+^ Current, Fast/Leak)

$\frac{{dm}_{Ca}}{dt}= \frac{1}{\tau_{mCa}}(m_{Ca}-m_{\infty Ca})$ 14. (T-type Ca^2+^ Current)

$\frac{{dh}_{Ca}}{dt}= \frac{1}{\tau_{hCa}}(h_{Ca}-h_{\infty Ca})$ 15. (T-type Ca^2+^ Current)

$\frac{{dm}_{KCa}}{dt}= \frac{1}{\tau_{mKCa}}(m_{KCa}-m_{\infty KCa})$ 16. (Ca^2+^-dependent K^+^ Current)

$\frac{{dm}_{CAN}}{dt}= \frac{1}{\tau_{mCAN}}(m_{CAN}-m_{\infty CAN})$ 17. (Ca^2+^-dependent Nonspecific Cation Current)

$\alpha_{m}=\frac{0.32\left( 54-V_{m} \right)}{(1-\exp\left[ -\frac{V_{m}+54}{4} \right])}$ 18. (Na^+^/K^+^ Current, Fast/Leak)

$\beta_{m}=\frac{0.28\left( V_{m+27} \right)}{(\exp\left[ \frac{V_{m}+27}{5} \right]-1)}$ 19. (Na^+^/K^+^ Current, Fast/Leak)

$\alpha_{h}=0.128exp[-\frac{50+V_{m}}{18}]$ 20. (Na^+^/K^+^ Current, Fast/Leak)

$\beta_{h}=\frac{4}{(1+\exp\left[ -\frac{V_{m}+27}{5} \right])}$ 21. (Na^+^/K^+^ Current, Fast/Leak)

$\alpha_{j}=\frac{0.032\left( V_{m}+52 \right)}{(1-\exp\left[ -\frac{V_{m}+52}{5} \right])}$ 22. (Na^+^/K^+^ Current, Fast/Leak)

$\beta_{j}= 0.5exp[-\frac{V_{m}+57}{40}]$ 23. (Na^+^/K^+^ Current, Fast/Leak)

$m_{\infty Ca}=\frac{1}{(1+\exp\left[ -\frac{V_{m}+52}{7.4} \right])}$ 24. (T-type Ca^2+^ Current)

$h_{\infty Ca}=\frac{1}{(1+\exp\left[ \frac{V_{m}+80}{5} \right])}$ 25. (T-type Ca^2+^ Current)

$\tau_{mCa}=0.44+ \frac{0.15}{(\exp\left[ \frac{V_{m}+27}{10} \right]+\exp\left[ -\frac{V_{m}+102}{15} \right])}$ 26. (T-type Ca^2+^ Current)

$\tau_{hCa}=22.7+ \frac{0.27}{(\exp\left[ \frac{V_{m}+48}{4} \right]+\exp\left[ -\frac{V_{m}+407}{50} \right])}$ 27. (T-type Ca^2+^ Current)

$\tau_{mKCa}=\frac{1}{(48\left[ {Ca}^{2+} \right]_{i}^{2}+0.03)}$ 28. (Ca^2+^-dependent K^+^ Current)

$m_{\infty KCa}=\frac{48\left[ {Ca}^{2+} \right]_{i}^{2}}{(48\left[ {Ca}^{2+} \right]_{i}^{2}+0.03)}$ 29. (Ca^2+^-dependent K^+^ Current)

$\tau_{mCAN}=\frac{1}{(20\left[ {Ca}^{2+} \right]_{i}^{2}+0.002)}$ 30. (Ca^2+^-dependent Nonspecific Cation Current)

$m_{\infty CAN}=\frac{20\left[ {Ca}^{2+} \right]_{i}^{2}}{(20\left[ {Ca}^{2+} \right]_{i}^{2}+0.002)}$ 31. (Ca^2+^-dependent Nonspecific Cation Current)

**Dynamic Ion Reversal Potentials –**

$E_{Na}\boldsymbol{=}26.64 ln(\frac{\left[ {Na}^{+} \right]_{o}}{\left[ {Na}^{+} \right]_{i}})$ 32. (Na^+^ Reversal Potential)

$E_{K}\boldsymbol{=}26.64 ln(\frac{\left[ K^{+} \right]_{o}}{\left[ K^{+} \right]_{i}})$ 33. (K^+^ Reversal Potential)

$E_{Cl}\boldsymbol{=}26.64 ln(\frac{\left[ {Cl}^{-} \right]_{i}}{\left[ {Cl}^{-} \right]_{o}})$ 34. (Cl^-^ Reversal Potential)

$E_{Ca}\boldsymbol{=}26.64 ln(\frac{\left[ {Ca}^{2+} \right]_{o}}{\left[ {Ca}^{2+} \right]_{i}})$ 35. (Ca^2+^ Reversal Potential)

**Dynamic Number of Each Ion (Inside/Outside the Cell)–**

$\frac{dN{Na}_{o}^{+}}{dt}=\tau V_{o}(\gamma_{con}I_{Na}+{3\gamma}_{con}I_{NCX}+\gamma_{con}I_{NaCAN}+3I_{pump}$ 36.

$\frac{dN{Na}_{i}^{+}}{dt}=\tau V_{i}(-\gamma_{con}I_{Na}-{3\gamma}_{con}I_{NCX}-\gamma_{con}I_{NaCAN}-3I_{pump}$ 37.

$\frac{dNK_{o}^{+}}{dt}=\tau V_{o}(\gamma_{con}I_{K}+\gamma_{con}I_{KCa}+\gamma_{con}I_{KCAN}-2I_{pump}+ I_{kcc2}- I_{diff}- I_{glia}- {2I}_{gliapump})$ 38.

$\frac{dNK_{i}^{+}}{dt}=\tau V_{i}(-\gamma_{con}I_{K}-\gamma_{con}I_{KCa}-\gamma_{con}I_{KCAN}+2I_{pump}- I_{kcc2})$ 39.

$\frac{dN{Cl}_{o}^{-}}{dt}=\tau V_{o}({-\gamma}_{con}I_{ClL}+ I_{kcc2} )$ 40.

$\frac{dN{Cl}_{i}^{-}}{dt}=\tau V_{i}(\gamma_{con}I_{ClL}- I_{kcc2} )$ 41.

$\frac{dN{Ca}_{i}^{2+}}{dt}=\tau V_{i}(-\gamma_{con}I_{T}+\gamma_{con}I_{NCX}-{Ca}_{absp})$ 42.

**Conversion to Dynamic Ion Concentration (Inside/Outside the Cell) –**

${[{Na}^{+}]}_{o}\boldsymbol{=}\frac{N{Na}_{o}}{V_{o}}$ 43.

${[{Na}^{+}]}_{i}\boldsymbol{=}\frac{N{Na}_{i}}{V_{i}}$ 44.

${[K^{+}]}_{o}\boldsymbol{=}\frac{NK_{o}}{V_{o}}$ 45.

${[K^{+}]}_{i}\boldsymbol{=}\frac{NK_{i}}{V_{i}}$ 46.

${[{Cl}^{-}]}_{o}\boldsymbol{=}\frac{N{Cl}_{o}}{V_{o}}$ 47.

${[{Cl}^{-}]}_{i}\boldsymbol{=}\frac{N{Cl}_{i}}{V_{i}}$ 48.

${[{Ca}^{2+}]}_{i}\boldsymbol{=}\frac{N{Ca}_{i}}{V_{i}}$ 49.

**Pumps and Cotransporters –**

$I_{pump}=(\frac{p_{max}}{1+\exp\left[ \frac{25-\left[ {Na}^{+} \right]_{i}}{3} \right]})(\frac{1}{1+\exp\left[ 5.5-\left[ K^{+} \right]_{o} \right]})$ 50. (neuronal Na^+^/K^+^-ATPase)

$I_{gliapump}=(\frac{1}{3})(\frac{p_{max}}{1+\exp\left[ \frac{25-\left[ {Na}^{+} \right]_{gi}}{3} \right]})(\frac{1}{1+\exp\left[ 5.5-\left[ K^{+} \right]_{o} \right]})$ 51. (glial Na^+^/K^+^-ATPase)

$I_{glia}=(\frac{G_{glia}}{1+\exp\left[ \frac{18-\left[ K^{+} \right]_{o}}{2.5} \right]})$ 52. (glial K^+^ uptake)

$I_{diff}=\epsilon_{k}(\left[ K^{+} \right]_{o}-\left[ K^{+} \right]_{bath})$ 53. (diffusion of K^+^ between extracellular space and vasculature)

$I_{kcc2}\boldsymbol{=}U_{kcc2} ln(\frac{{\left[ K^{+} \right]_{i}\left[ {Cl}^{-} \right]}_{i}}{{\left[ K^{+} \right]_{o}\left[ {Cl}^{-} \right]}_{o}})$ 54. (K^+^/Cl^-^ cotransporter, KCC2)

**Table 1 - Parameters**

| *Parameter* | *Value/Units* | *Description/Source* |
| --- | --- | --- |
| $C_{M}$ | 1.0 uF/cm^2^ | **Membrane Capacitance:** Destexhe et al. 1994, Wei et al., 2014, Toglia and Ullah 2019 |
| $g_{Na}$ | 30.0 μA/(mV*cm^2^) | **Na^+^ fast current conductance:** Wei et al., 2014, Toglia and Ullah 2019 |
| $g_{K}$ | 25.0 μA/(mV*cm^2^) | **K^+^ fast current conductance:** Wei et al., 2014, Toglia and Ullah 2019 |
| $g_{NaL}$ | 0.0247 μA/(mV*cm^2^) | **Na^+^ leak conductance:** Toglia and Ullah 2019 |
| $g_{KL}$ | 0.1855 μA/(mV*cm^2^)  0.22062 μA/(mV*cm^2^) | **K^+^ leak conductance:** Calculated/Adjusted |
| $g_{ClL}$ | 0.49 uA/(mV*cm^2^) | **Cl^-^ leak conductance:** Calculated/Adjusted |
| $g_{TCa}$ | 1.75 uA/(mV*cm^2^) | **T-type Ca^2+^ current conductance:** Destexhe et al. 1994 |
| $g_{KCa}$ | 10.00 uA/(mV*cm^2^) | **Ca^2+^-dependent K^+^ current conductance:** Destexhe et al. 1994 |
| $g_{CAN}$ | 0.25 uA/(mV*cm^2^) | **Ca^2+^-dependent nonspecific cation current conductance:** Destexhe et al. 1994 |
| $g_{NaCAN}$ | 0.124 uA/(mV*cm^2^) | **Ca^2+^-dependent nonspecific cation current conductance, Na^+^ part:** calculated from Destexhe et al. 1994 |
| $g_{KCAN}$ | 0.126 uA/(mV*cm^2^) | **Ca^2+^-dependent nonspecific cation current conductance, K^+^ part:** calculated from Destexhe et al. 1994 |
| $I_{NCXmax}$ | 1600 pA/pF | **Maximum rate of Na^+^/K^+^ exchanger:** Courtemanche et al. 1998, Kager et al. 2007 |
| $p_{max}$ | 1.25 mM/s | **Maximum rate of Na^+^/K^+^/ATP pump:** Wei et al. 2014 |
| $U_{kcc2}$ | 0.3 mM/s | **Maximum rate of K^+^/Cl^-^ cotransporter, KCC2:** Toglia and Ullah 2019 |
| $G_{glia}$ | 8.0 mM/s | **Maximum rate of glial K^+^ uptake:** Wei et al. 2014 |
| $\epsilon_{k}$ | 0.33 1/s | **Maximum rate of diffusion of K^+^ between extracellular space and vasculature:** Wei et al. 2014 |
| $\left[ K^{+} \right]_{bath}$ | 4.0 mM | **K^+^ bath concentration:** Wei et al. 2014 |
| $\left[ {Na}^{+} \right]_{gi}$ | 18.0 mM | **Na^+^ concentration in glia:** Toglia and Ullah 2019 |
| $\tau$ | 0.001 | **Conversion parameter, from ms to s:** Toglia and Ullah 2019 |
| $V_{i}$ | 1.4368 x 10^-15^ m^3^ | **Intracellular volume:** Wei et al. 2014 |
| $V_{o}$ | 2.05257 x 10^-16^ m^3^ | **Extracellular volume:** Wei et al. 2014 |
| $\gamma_{con}$ | 0.0445 | **Conversion parameter, from current in μA/cm^2^ to concentration in mM/s:** Wei et al. 2014 |
| $E_{CAN}$ | -20.0 mV | **Resting potential for Ca^2+^-dependent nonspecific cation current:** Destexhe et al. 1994 |
| $K_{m,Na}$ | 87.5 mM | **[Na^+^]_o_ half-saturation constant for Na^+^/K^+^ exchanger:** Courtemanche et al. 1998, Kager et al. 2007 |
| $K_{m,Ca}$ | 1.38 mM | **[Ca^2+^]_o_ half-saturation constant for Na^+^/K^+^ exchanger:** Courtemanche et al. 1998, Kager et al. 2007 |
| $k_{sat}$ | 0.1 | **Saturation factor for Na^+^/K^+^ exchanger:** Courtemanche et al. 1998, Kager et al. 2007 |
| $\gamma$ | 0.35 | **Voltage dependence parameter for Na^+^/K^+^ exchanger:** Courtemanche et al. 1998, Kager et al. 2007 |
| R | 8.3143 J/(K x mol) | **Gas constant** |
| T | 310 K | **Temperature:** Courtemanche et al. 1998, Kager et al. 2007 |
| F | 96.4867 C/mmol | **Faraday’s Constant:** Courtemanche et al. 1998, Kager et al. 2007 |

**Table 2 – Initial Values**

| *Initial Value* | *Value/Units* | *Description/Source* |
| --- | --- | --- |
| ${[{Na}^{+}]}_{o}$ | 144.0 mM | **Initial extracellular Na^+^ concentration:** Wei et al. 2014 |
| ${[{Na}^{+}]}_{i}$ | 18.0 mM | **Initial intracellular Na^+^ concentration:** Wei et al. 2014 |
| ${[K^{+}]}_{o}$ | 4.0 mM | **Initial extracellular K^+^ concentration:** Wei et al. 2014 |
| ${[K^{+}]}_{i}$ | 140.0 mM | **Initial intracellular K^+^ concentration:** Wei et al. 2014 |
| ${[{Cl}^{-}]}_{o}$ | 130.0 mM | **Initial extracellular Cl^-^ concentration:** Wei et al. 2014 |
| ${[{Cl}^{-}]}_{i}$ | 6.0 mM | **Initial intracellular Cl^-^ concentration:** Wei et al. 2014 |
| ${[{Ca}^{2+}]}_{o}$ | 2.0 mM | **Initial extracellular Ca^2+^ concentration:** Destexhe et al. 1994 |
| ${[{Ca}^{2+}]}_{i}$ | 2.4 x 10^-4^ mM | **Initial intracellular Ca^2+^ concentration:** Destexhe et al. 1994 |
| $m_{H}$ | 0.0036 | Wei et al. 2014 |
| $j_{H}$ | 0.0122 | Wei et al. 2014 |
| $h_{H}$ | 0.9992 | Wei et al. 2014 |
| $m_{Ca}$ | 0.0 | Destexhe et al. 1994 |
| $h_{Ca}$ | 1.0 | Destexhe et al. 1994 |
| $m_{KCa}$ | 0.0 | Destexhe et al. 1994 |
| $m_{CAN}$ | 0.0 | Destexhe et al. 1994 |

1. I_NaCAN_ + I_KCAN_ = I_CAN_ [↑](#footnote-ref-1)
