# Supplementary material for: Computational model of alpha 7 nicotinic acetylcholine receptor in thalamic reticular nucleus neurons and their involvement in network states
Source: PLoS One. 2025 Aug 29;20(8):e0330635. doi: 10.1371/journal.pone.0330635 (PMC12396715; doi:10.1371/journal.pone.0330635)
Supplement: Attachment — Submitted filename: Reviewer_response_FINAL.docx [file pone.0330635.s003.docx]

PONE-D-25-14714

Computational model of alpha 7 nicotinic acetylcholine receptor in thalamic reticular nucleus neurons and their involvement in network states

Reviewer's Responses to Questions

Review Comments to the Author

Please use the space provided to explain your answers to the questions above. You may also include additional comments for the author, including concerns about dual publication, research ethics, or publication ethics. (Please upload your review as an attachment if it exceeds 20,000 characters)

Reviewer #1: The manuscript by Ullah et al. presents a single-compartment Hodgkin-Huxley (HH) model of a TRN neuron incorporated into a simplified thalamic network model, aiming to demonstrate that alpha7 nAChR activity modulates TC oscillations. Overall the scientific questions are clearly defined and the methodological approach is correct. However, there are several major issues that need to be addressed before the manuscript can be accepted for publication.

Here are my comments:

MAJOR CONCERNS

1) The Materials and Methods section lacks sufficient detail, and the overall presentation of the results is difficult to follow. For example, the current input (Iapp) in the first set of equations is not explained, and the inclusion of the Nernst equation seems redundant and can be omitted to include other fundamental elements (see below). The authors should provide a more thorough and clear presentation of the materials and methods.

We appreciate the reviewer’s comments. We have added the details regarding the input current (Iapp) in the text. Specifically, Iapp represents an externally applied current stimulus that initiates network activity or simulates synaptic input. Its amplitude and timing are varied based on the experimental conditions being modeled, which is described in the mansucript.

Additionally, we have moved the Nernst equations to Supplementary Tables 1 and 2 for clarity. The revised text now reads: Details of the dynamic reversal potential for Na^+^ (E_NA_), K^+^ (E_K_), Cl^-^ (E_Cl_), and Ca^2+^ (E_Ca_), as well as the Ca2+ dynamics, ion concentration equations, and parameter values, are provided in Supplementary Tables 1 and 2. Differential equations were solved using ode15s in MATLAB (MathWorks, Natick, MA).

A highly detailed description of the Neural Network model, experimental parameters, and figure components is now presented in Supplementary Material.

2) The authors claim to have constructed a neural network consisting of a single TRN neuron and an unspecified number of TC relay neurons. Can the dynamic description of an inhibitory rebound excitation be strictly considered a neural network model? Please comment more about your assumptions.

We appreciate the reviewer’s question. Our use of the term “neural network model” refers to a minimalistic network rather than a large-scale spiking network involving multiple interconnected neurons. Specifically, our model comprises a single thalamic reticular nucleus (TRN) neuron interacting with a simplified representation of thalamocortical (TC) relay activity through a phenomenological feedback mechanism designed to capture the dynamics of inhibitory rebound excitation. This is now well described in the Methods and detailed in the Supplement section of the manuscript.

Specifically, the rebound excitation is modeled not through an explicit population of TC neurons but as a depolarizing current input with a recovery variable that obeys first-order kinetics. This formulation was chosen for computational efficiency and clarity in exploring parameter-dependent reverberation behaviors. While this does not constitute a full-scale network with multiple neuron instances and synaptic weights, it captures essential dynamic interactions between inhibition and excitation that are well-documented in thalamic circuitry.

Thus, we agree that this model may not qualify as a neural network in the traditional sense of multiple neuron populations; however, it is a computational abstraction of a recurrent loop that simulates the interplay between TRN inhibition and TC rebound. We have clarified this point in the revised text and now refer to it as a "minimal network model" to more accurately reflect its scope and structure.

3) In the "Neural Network Model" section, the authors reports: "........Finally, we incorporated a parameter controlling the strength of a potassium leak current modeling adenosine activated channels as a means to control the wakefulness of the network. This final parameter affects the resting potential, thus playing a key role in toggling between T-channel mediated tonic and burst firing.........."
However, the name of this parameter and its modulation range is neither mentioned in the main text nor in the supplemental material. Please provide a complete description of the model, including this missing information. Since the work is entirely based on modeling, it is essential that all model parameters are fully described.

We thank the reviewer for pointing out this oversight. The parameter is simply the leak conductance, G_KL_ already introduced in the model. We have made this more explicit as well as stating the values used to establish resting membrane potentials consistent with wake and sleep states. A detailed description of the model is presented in Supplementary Material.

4) In the Results section, under "Neural Network Dynamics and the Sleep Axis," a description of the network is provided. This network description should be included earlier in the Methods section, which currently lacks essential information necessary to understand how the network is conceptualized and implemented. Most importantly the explanation of the sleep axis isn't clear. A dedicated section should be included in the Methods, outlining this aspect along with an explanation of the criteria used to assess the impact of parameters on the network's behavior.

We agree with the reviewer that this discussion should be moved up in the text. We have moved the description of the model to the methods as well as a more detailed explanation of the sleep-axis as well as the histograms and correlation analysis as well. A detailed description is presented in Supplementary Material. It is noteworthy, that we attempt to argue that the progression of the parameters is based on what should be expected from wake-sleep inducing mechanisms and further argue in the results that the parameters produce dynamics that appear to progress from wake to sleep like along the sleep-axis parameter.

5) The explanation of the Results and the related figures are not sufficiently clear. Figure 7 and 8 are poorly commented.
Fig.7: How is the spectral analysis performed? What does the count plot represent? The colorbar of the heatmap is missing together with an adeguate description of plot axes and parameter changes. What do the black circles represent? Indicating the decreasing of the alpha7 with a black arrow is not straightforward.
The authors write "White horizontal rectangles intersect the low adenosine regions of the normal, bottom, and 50% expression, top, in Fig. 7A", where are the white horizontal rectangles in the figure?
Fig 8: How is the cross-correlation performed?

We thank the referee for pointing out these inadequacies. We have bolstered our explanation of the histograms in the Methods section and accompanying Supplementary Material section. We did not include a color bar because we are only interested in displaying the relative frequencies of the rates. We would prefer to not take up the space to show the relative scale but will certainly include one if the reviewer deems it necessary. The circles and ovals are to indicate the inter and intra-burst rates for the sleep like states. We have added text to the results to describe these states.

Sorry for the confusion, the black arrow was not intended to indicate decreasing alpha7. Alpha7 is set at different values (0, 50,75, 90, 100, 110, 125%) for each of the seven columns in Fig 7C. The black arrow indicates the shifting up of frequencies while the alpha7 shifts to the left. This is now clarified in the Result section.

Apologies, the white horizontal rectangles are in figure 8 not 7. This is now corrected.

The cross-correlation was performed using Matlab’s corrcoef.m function that computes the Pearson coefficient for two arrays. It is equivalent to the zero-lag value for a standard cross correlation function. We tested to make sure we got the same result using our go-to cross correlation algorithm but used the Matlab function for ease of use.

6) "Here, the dynamics between wake, the first horizontal third, differs from the sleep states, middle and right thirds, in several remarkable ways." This sentence is impossible for me to decipher, and the significant differences are not explicitly discussed.

We have significantly revised this section and expanded on our description of the dynamical states in the revised manuscript.

7) Does the model effectively replicate the accelerando-decelerando burst activity observed experimentally? This evidence should be thoroughly highlighted and discussed in the Results section, rather than being confined to the Discussion.

We thank the reviewer for catching this important oversight. Yes, our model effectively replicates the accelerando–decelerando burst activity observed experimentally. As shown in Fig. 2B, the burst response triggered by hyperpolarizing current injection exhibits a progressive increase in inter-spike frequency (accelerando phase) followed by a gradual slowing down (decelerando phase) within the burst. This temporal structure closely mirrors the intrinsic firing behavior of thalamic reticular neurons reported by Fuentealba and Steriade [69], who demonstrated that thalamic oscillations modulate membrane properties to produce such bursting. Notably, our model reproduces this dynamic without requiring parameter adjustments beyond physiological constraints, underscoring its validity in capturing TRN neuron activity. Accordingly, this result is now emphasized in the Results section to highlight the model's fidelity to known experimental findings.

Specific text added to the revision: “This transition from bursting to spiking is clearly illustrated in the zoomed-in portion of the action potential trace (Fig. 2B), exhibiting a bursting behavior similar to that reported by Fuentealba et al. [69], which confirms the characteristic accelerando–decelerando bursting activity. Figu 2B has been added to clearly show the accelerando and decelerando phases.

MINOR

1) There are several minor typos that need to be corrected. For example:

"nonselective"

Fixed

"INCXusing"

Fixed

"activitybursts"

Fixed

A careful review of the English is highly recommended.

The manuscript has been revised for language throughout.

2) The legend of Fig.6 depicts the neural network but the legend doesn't explain all the elements of the figure (what is BF, LC, RN, BS?). Once that the figure layout has been improved please check carefully the legend description that must match the figure content.

Fixed
